# Identification of miRNA biomarkers for stomach adenocarcinoma

**DOI:** 10.1186/s12859-022-04719-6

**Published:** 2022-05-16

**Authors:** Hao Qian, Nanxue Cui, Qiao Zhou, Shihai Zhang

**Affiliations:** Department of Anesthesiology, Union Hospital, Tongji Medical College, Huazhong University of Science and Technology, Wuhan, 430000 Hubei Province China

**Keywords:** TCGA, STAD, miRNA, Bioinformatics, Prognostic model

## Abstract

**Background:**

Stomach adenocarcinoma (STAD) is a common malignant tumor in the world and its prognosis is poor, miRNA plays a role mainly by influencing the expression of mRNAs, and participates in the occurrence and development of tumors. However, reliable miRNA prognostic models for stomach adenocarcinoma remain to be identified.

**Results:**

Using the data from the Cancer Genome Atlas (TCGA), a prognostic model of stomach adenocarcinoma was established including tumor stage and expression levels of 4 miRNAs (hsa-miR-379-3p, hsa-miR-2681-3p, hsa-miR-6499-5p and hsa-miR-6807-3p). A total of 50 ultimate target genes of these miRNAs were obtained through prediction. Enrichment analysis revealed that target genes were mainly concentrated in neural function and TGF-β and FoxO signaling pathways. Survival analysis showed that three model miRNAs (hsa-miR-379-3p, hsa-miR-2681-3p and hsa-miR-6807-3p) and five final target genes (*DLC1*, *LRFN5*, *NOVA1*, *POU3F2* and *PRICKLE2*) were associated with the patient's overall survival outcome.

**Conclusions:**

We used bioinformatics methods to screen new prognostic miRNA markers from TCGA and established a prognostic model of STAD, so as to provide a basis for the diagnosis, prognosis, and treatment of STAD in the future.

**Supplementary Information:**

The online version contains supplementary material available at 10.1186/s12859-022-04719-6.

## Background

Stomach cancer is a common malignant tumor in the world. It ranks sixth in incidence and third in mortality in global cancer statistics in 2020 [1], while stomach adenocarcinoma (STAD) is the most prevalent pathological type of stomach cancer [2]. Although with the development of research on the mechanism of stomach cancer, the treatment of gastric cancer has gradually diversified. In addition to surgical treatment, radiotherapy and chemotherapy, molecular targeted therapy has also begun to emerge, but the survival rate of stomach cancer is still very low, mainly because the patients with stomach cancer is usually diagnosed in the middle and late stage, so the early recognition of stomach cancer is particularly important.

**Fig. 1:**
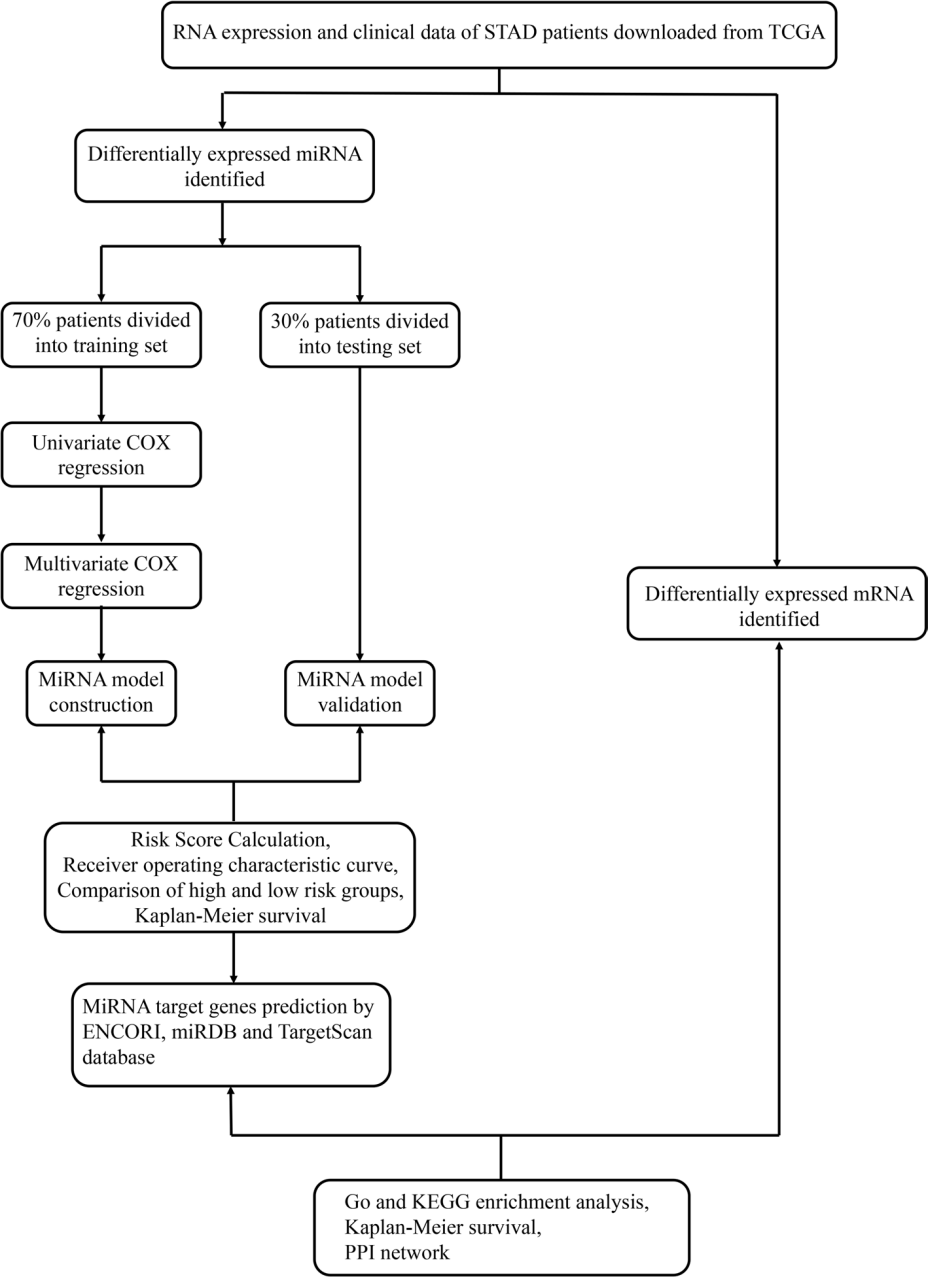
The flowchart for construction and validation of the miRNA biomarkers

MiRNA is a type of non-coding RNA (nc RNA), which plays a role mainly by influencing the expression of mRNAs, and participates in many important life processes including the occurrence and development of tumors [3]. A study showed that miR-4521 plays a role in regulating gastric cancer metastasis and tumor cell hypoxia response [4]. Furthermore, miR-125b-5p, miR-196a-5p, miR-1-3p and miR-149-5p have also been confirmed as non-invasive indicators for clinical diagnosis [5]. However, due to the time-consuming and labor-intensive exploration of the role of miRNAs in the occurrence and development of diseases through experimental methods, the use of computational methods to predict the relationship between miRNAs and diseases has gradually become an effective complement. For example, the application of computational model of inductive matrix completion [6], matrix decomposition and heterogeneous graph inference [7] and neighborhood constraint matrix completion [8] in the prediction of miRNA-disease association. Nevertheless, there is currently no method to evaluate the overall survival of STAD patients and the possibility of targeted therapy through miRNAs. Therefore, we embarked on this research (Fig. 1) to identify the reliable prognostic model in STAD patients by using miRNA, mRNA and clinical information obtained from the cancer genome Atlas (TCGA), and to provide new insights for future targeted therapies.

## Results

### Differentially expressed mRNAs and miRNAs

**Fig. 2:**
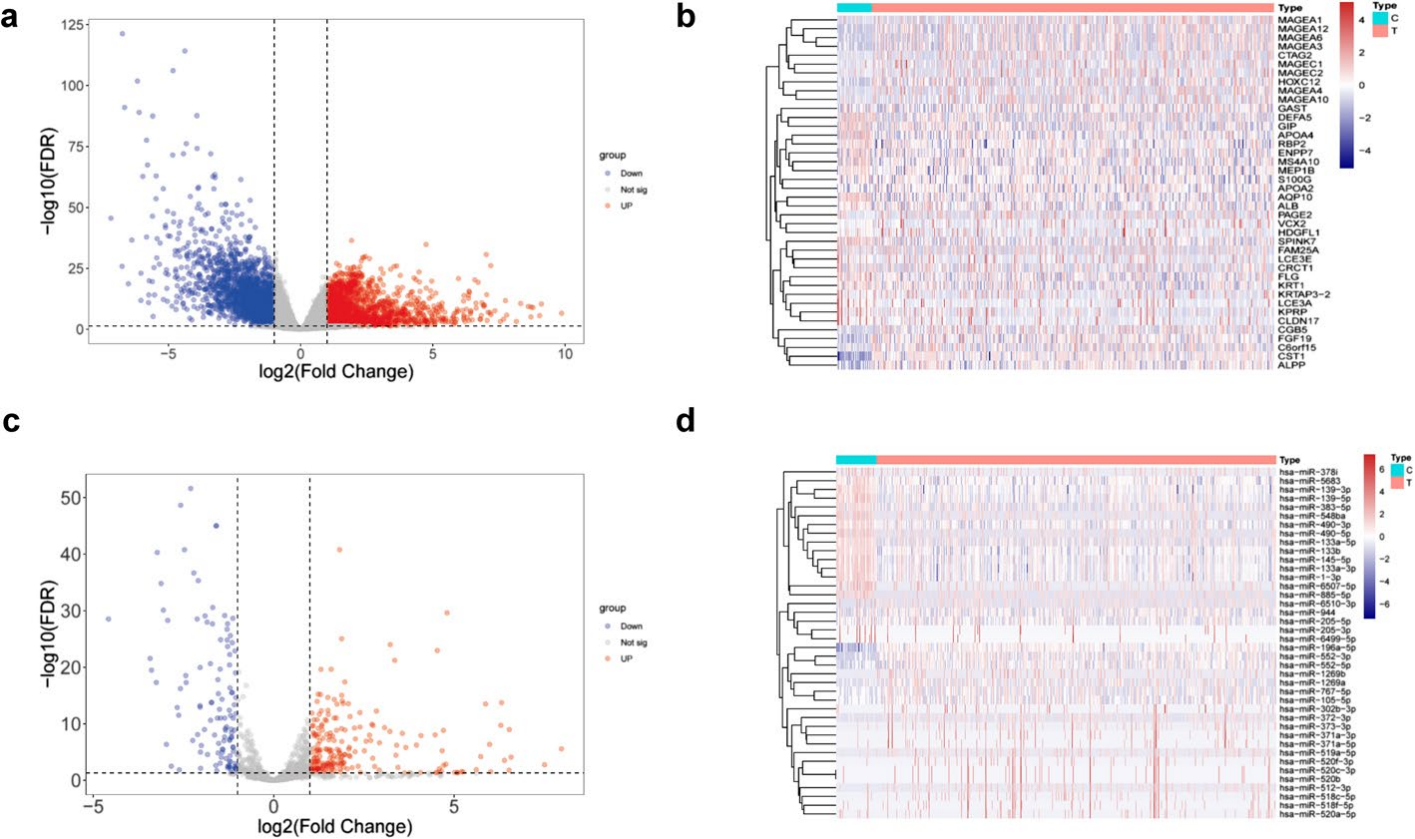
The volcano plot of differentially expressed mRNA and miRNA and the heat map of the top 20 up-regulated and down-regulated mRNA and miRNA. **a**, **b** mRNAs; **c**, **d** miRNAs

Through the following method of obtaining differential genes, we finally obtained 4362 differently expressed mRNAs, among which 2206 and 2156 mRNAs were upregulated and downregulated, respectively (Additional file 1: Table S1). 338 differential expression genes miRNAs were obtained, among which 221 and 117 miRNAs were upregulated and downregulated, respectively (Additional file 1: Table S2). The results of differently expressed mRNAs and miRNAs were displayed in volcano plots respectively (Fig. 2a, c), and the results of the top 20 upregulated and downregulated genes are displayed simultaneously in heatmaps (Fig. 2b, d).

### Prognostic models of miRNA

After the univariate COX analysis of the training set, tumor stage, hsa-miR-379-3p, hsa-miR-2681-3p, hsa-miR-6499-5p and hsa-miR-6807-3p were selected as five independent prognostic factors (Table 1 and Additional file 1: Table S3). Then a multivariate COX regression model was built through these five factors and factors were all found to be significant. The risk score of all patients can be calculated by following formula where h_0_(t) is the benchmark risk function, that is, the risk function at time t when all variables are zero, so h_0_(t) is a constant.

**Table 1:** Results of the univariate and multivariate Cox analyses of survival-related variables in the training set

Variables	Univariate			Multivariate		
	HR	CI95	p-value	HR	CI95	p-value
Age	0.62	0.35–1.10	0.103			
Gender	0.57	0.26–1.21	0.141			
Grade	1.54	0.88–2.70	0.129			
Stage	1.68	1.18–2.37	0.004	1.79	1.25–2.56	0.001
T	1.47	1.02–2.13	0.040			
N	1.30	0.99–1.71	0.059			
M	2.38	0.85–6.66	0.099			
hsa-miR-379-3p	1.72	1.17–2.53	0.006	1.77	1.15–2.72	0.010
hsa-miR-2681-3p	41.15	2.92–579.13	0.006	28.56	2.27–359.06	0.010
hsa-miR-6499-5p	3.14	1.34–7.37	0.009	4.84	2.07–11.30	0.0003
hsa-miR-6807-3p	4.97	1.88–13.13	0.001	6.41	2.15–19.13	0.0009

$$RiskScore={h}_{0}(t)\times exp(0.5825\times Stage+0.5688\times (expression\, of \,hsa-miR-379-3p)+3.3520\times \left(expression\,of\, hsa-miR-2681-3p\right)+1.5770\times \left(expression \,of\, hsa-miR-6499-5p\right)+1.8577\times \left(expression \,of \,hsa-miR-6807-3p\right)).$$

**Fig. 3:**
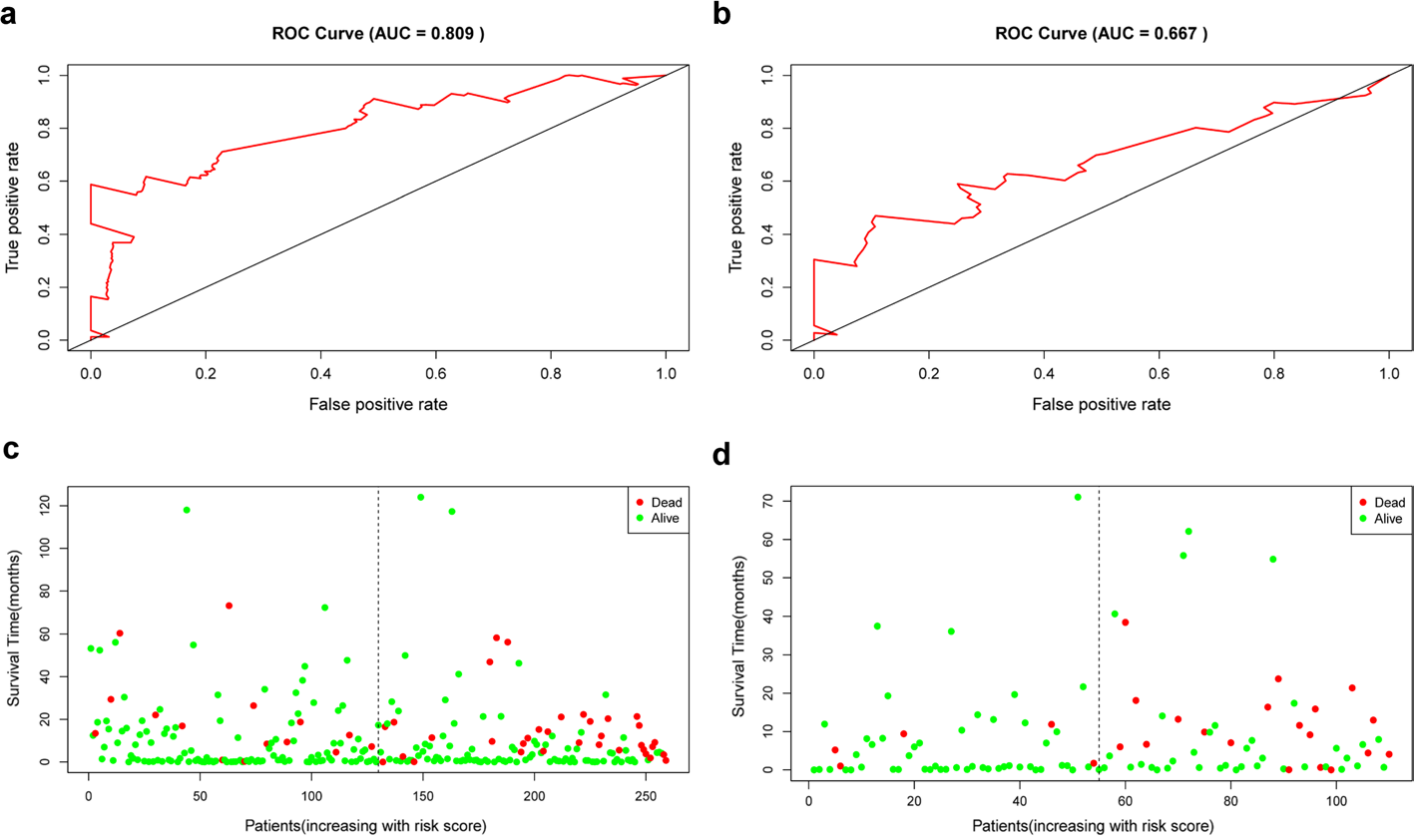
The ROC curve of the risk model for predicting the 5-year survival and the survival status plot in the training and the testing set. **a** ROC curve of training set; **b** ROC curve of testing set; **c** survival status plot of training set; **d** survival status plot of testing set

**Fig. 4:**
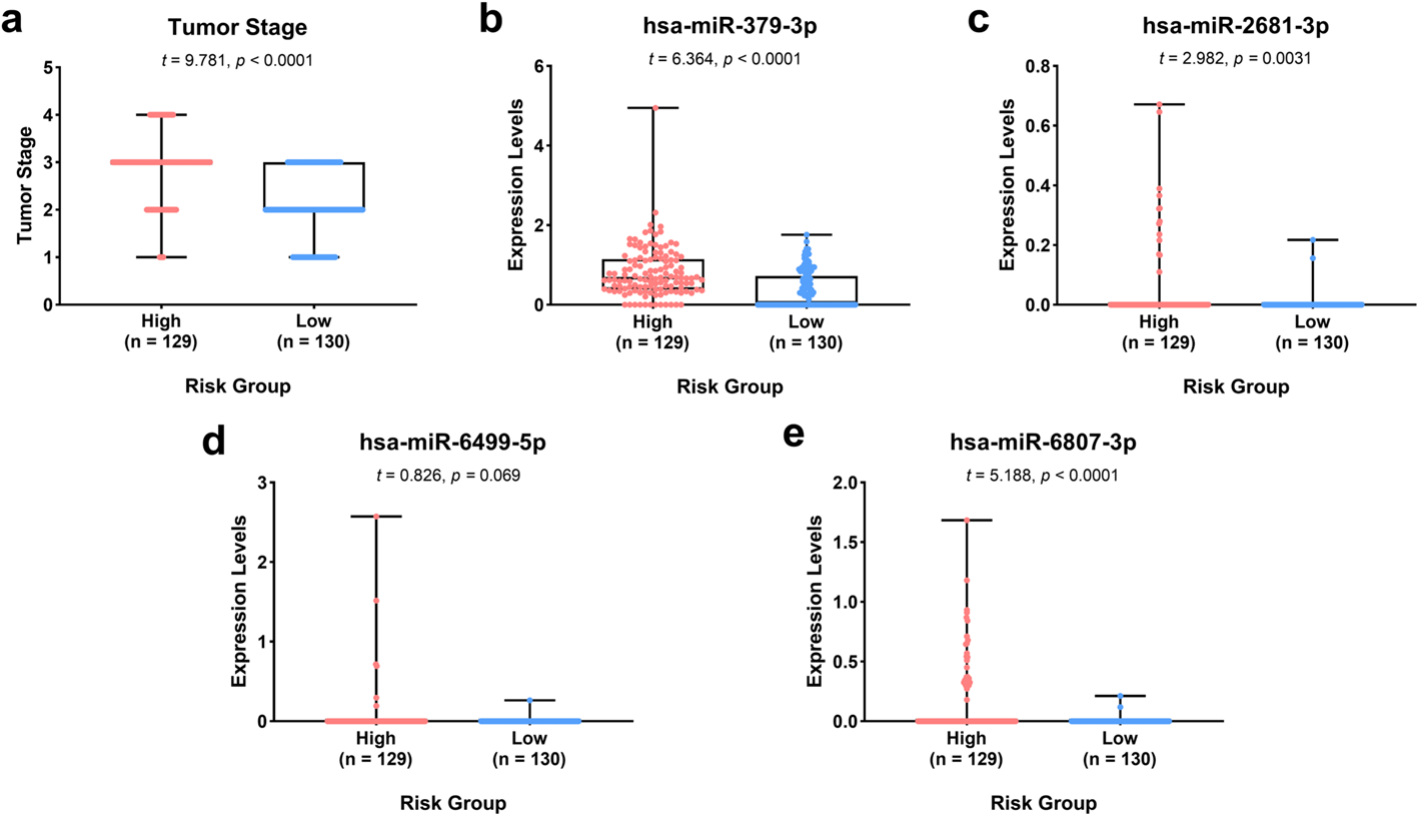
Boxplot plots showing the tumor stage and the expression levels of the 4 miRNA biomarkers in training set. **a** Tumor stage; **b** hsa-miR-379-3p; **c** hsa-miR-379-3p; **d** hsa-miR-6499-5p; **e** hsa-miR-6807-3p

**Fig. 5:**
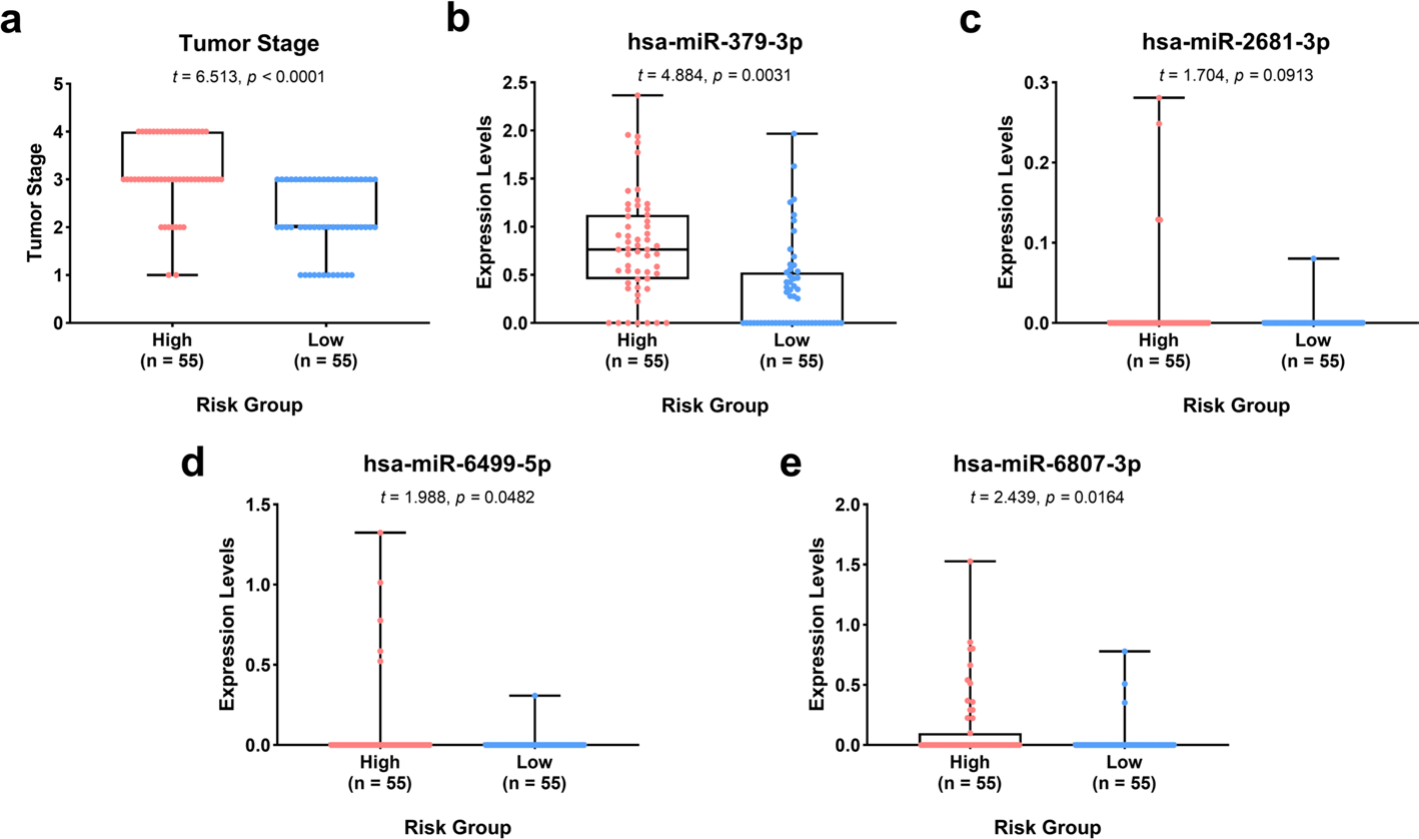
Boxplot plots showing the tumor stage and the expression levels of the 4 miRNA biomarkers in testing set. **a** Tumor stage; **b** hsa-miR-379-3p; **c** hsa-miR-379-3p; **d** hsa-miR-6499-5p; **e** hsa-miR-6807-3p

We drew Receiver operating characteristic (ROC) curve (Fig. 3a, b) to evaluate the accuracy of formula model. We found that the Area Under Curve (AUC) of training set and the testing set were 0.809 and 0.667, and another model assessment indicator C-index were 0.695 and 0.654, respectively, which means that the model has a moderate degree of accuracy. The survival status of the training set and the testing set were also showed in Fig. 3c, d. We also analyzed the expression levels of these four miRNAs and the tumor stage in the two groups (high-risk and low-risk) of training set and testing set. For training set, the tumor stages and the expression levels of hsa-miR-379-3p, hsa-miR-2681-3p, and hsa-miR-6807-3p were significantly different between the high and low risk groups, while the expression levels of hsa-miR-6499-5p did not have statistical difference (Fig. 4). For testing set, the tumor stages and the expression levels of hsa-miR-379-3p, hsa-miR-6499-5p and hsa-miR-6807-3p were significantly different between the high and low risk groups, while the expression levels of hsa-miR-2681-3p did not have statistical difference (Fig. 5).

### Survival analysis of miRNA

**Fig. 6:**
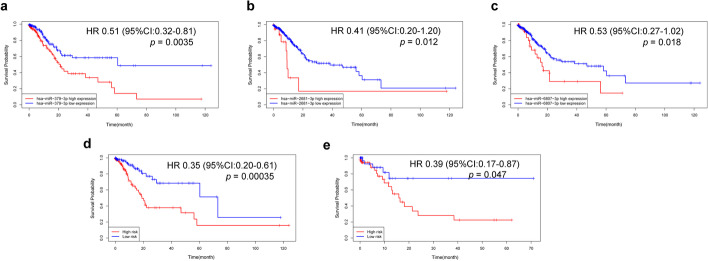
Survival analysis of 3 miRNA, the training and testing set using Kaplan–Meier analyses and the log rank tests. **a**–**c** miRNAs; **d** Training set; **e** Testing set

After Kaplan–Meier analyses and the log rank tests, we found that the expression of hsa-miR-379-3p, hsa-miR-2681-3p and hsa-miR-6807-3p (Fig. 6a–c) can affect the overall survival outcomes. In addition, we also evaluate the overall survival outcomes of high-risk score and low-risk score group in the training set and testing set (Fig. 6d, e) and found that the survival outcome of the low-risk score group was better than that of the high-risk score group.

### miRNA targets prediction

**Fig. 7:**
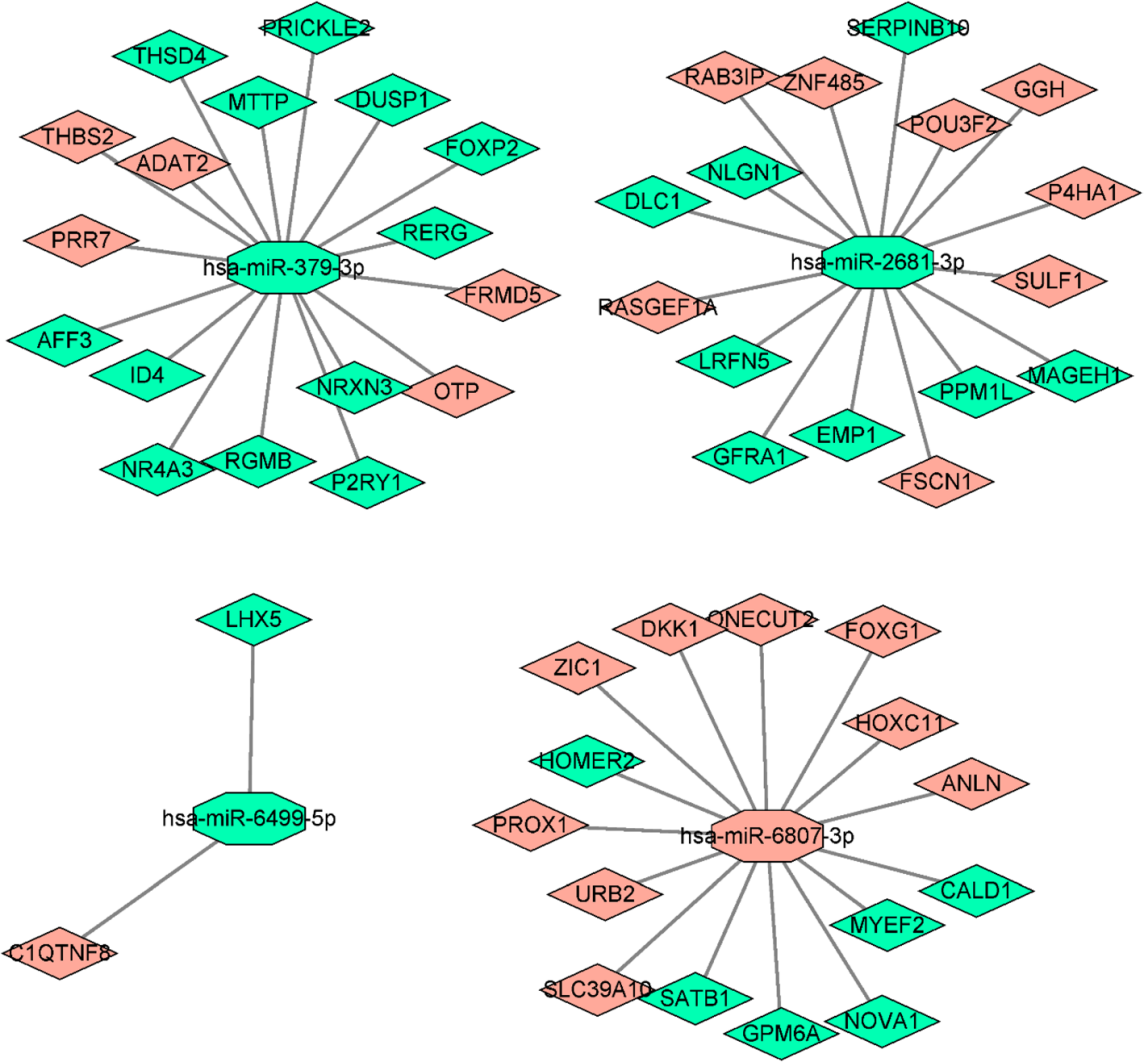
The connections between miRNAs and the ultimate target genes

**Fig. 8:**
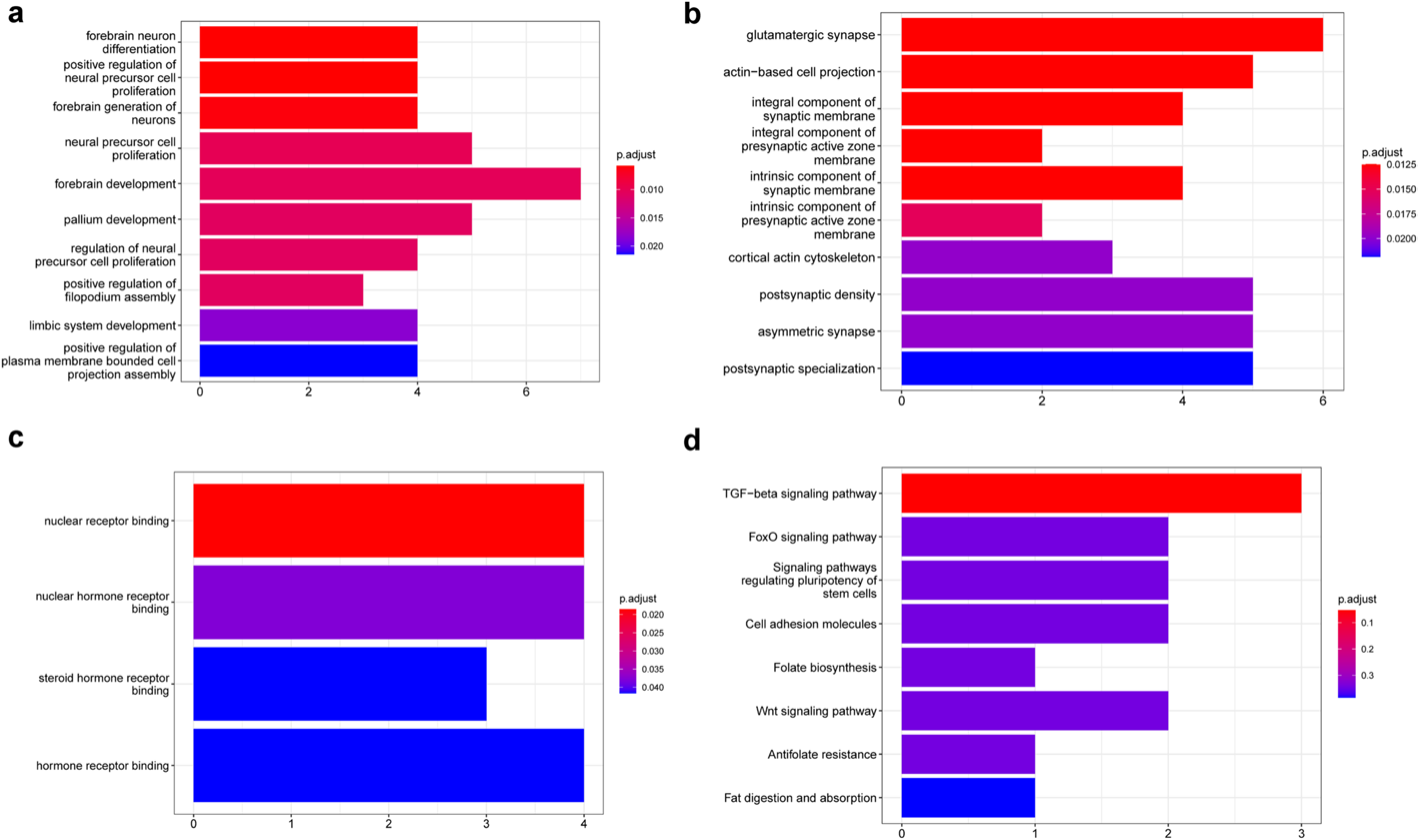
The GO and KEGG analysis of the ultimate target genes. **a** GO(BP); **b** GO(CC); **c** GO(MF); **d** KEGG

From the miRNA prediction database, we got 409 potential target genes, and 50 ultimate target genes (Additional file 1: Table S4) were finally obtained by intersection with differentially expressed mRNA. The connections between miRNAs and the ultimate target genes were visualized by Cytoscape software [9] as shown in Fig. 7, hsa-miR-379-3p, hsa-miR-2681-3p and hsa-miR-6807-3p had 17, 16 and 15 ultimate target genes, respectively, while hsa-miR-6499-5p had only 2 ultimate target genes. Gene Ontology (GO) and Kyoto Encyclopedia of Genes and Genomes (KEGG) pathway enrichment were utilized to evaluate the distribution of the ultimate target genes. The Biological Process (BP) results of Go displayed that the ultimate target genes concentrated in ‘forebrain neuron differentiation’, ‘positive regulation of neural precursor cell proliferation’ and ‘forebrain generation of neurons’ (Fig. 8a). Regarding Cellular Component (CC), the genes were enriched in ‘glutamatergic synapse’, ‘actin-based cell projection’ and ‘integral component of synaptic membrane’ (Fig. 8b). Under Molecular Function (MF), target genes were enriched in ‘nuclear receptor binding’, ‘nuclear hormone receptor binding’ and ‘steroid hormone receptor binding’ (Fig. 8c). In addition, the results of KEGG [10–12] revealed that target genes were enriched in TGF-beta signaling pathway and FoxO signaling pathway (Fig. 8d and Additional file 1: Table S5).

### Survival analysis of ultimate target genes

**Fig. 9:**
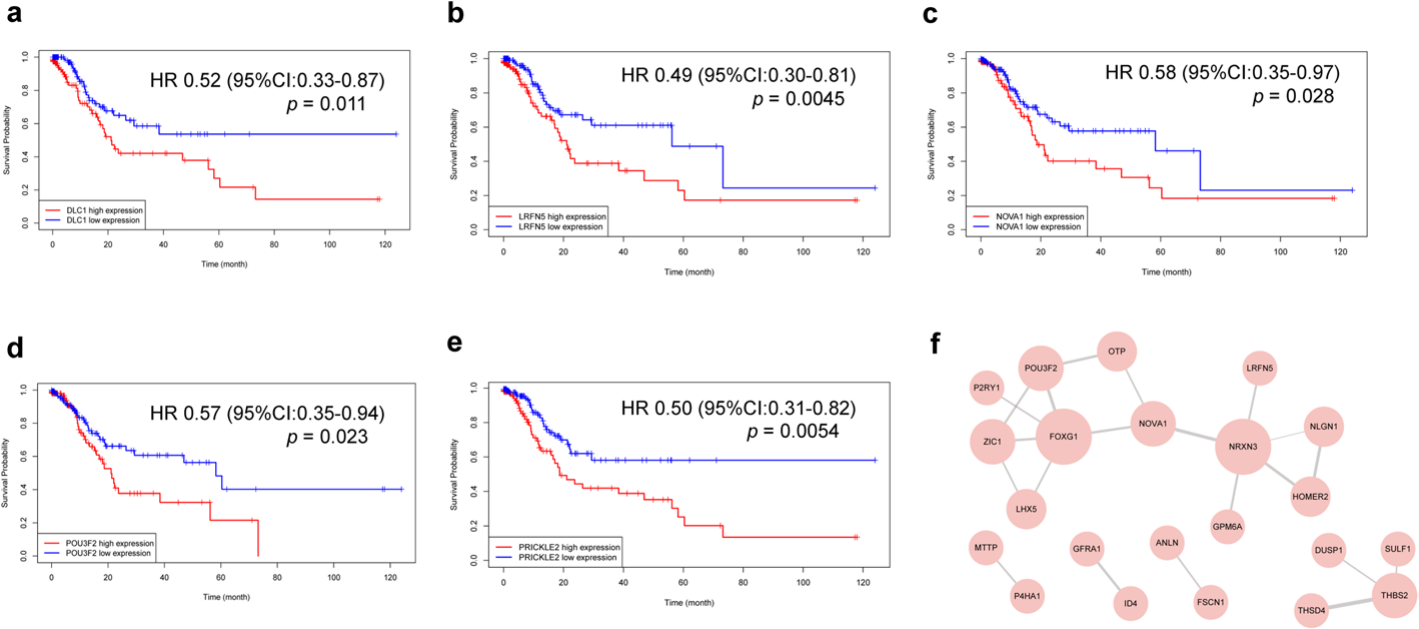
Survival analysis of ultimate target genes and the relationships between target proteins visualized by PPI. **a**–**e** mRNA; **f** PPI network

We analyzed the overall survival outcomes of the 50 ultimate target genes, and detected that the expression value of *DLC1*, *LRFN5*, *NOVA1*, *POU3F2* and *PRICKLE2* were positively related to the overall survival time (Fig. 9a–e). The results of protein–protein interaction network (PPI) revealed three hub genes (*FOXG3*, *NRXN3* and *NOVA1*) (Fig. 9f).

## Discussion

MiRNA is a type of nc RNA, which plays a role mainly by influencing the expression of mRNAs, and participates in many important life processes including the occurrence and development of tumors [3]. The function of miRNA, the interaction between miRNA and its target genes, and the relationship between miRNA and various diseases, especially cancers, have been widely studied by scientists. In addition to exploring the role of miRNAs through experimental research, computational models have gradually become an important means to identify the association between miRNAs and diseases [13]. Therefore, the analysis of miRNA patterns in different cancers may reveal the value of miRNA in cancer diagnosis, treatment, and prognosis evaluation. In this research, we used four mature miRNAs to construct a prognostic model which can make moderate predictions for the prognosis of STAD patients and provide new insights for treatment and diagnosis. The results of ROC curves of our models showed that the AUC of training set and testing set were 0.809 and 0.667, respectively. Indeed, when AUC value is greater than 0.7, it is generally considered that the model has good predictive ability. In this study, the AUC value of test set was 0.667, quite close to 0.7, so we believe that the model was of predictive power. A large gap between the AUCs of the training set and test set may be due to a relatively small sample size.

The four mature miRNAs including hsa-miR-379-3p, hsa-miR-2681-3p, hsa-miR-6499-5p, and hsa-miR-6807-3p, and tumor stage are the five prognostic factors for the prognosis of STAD patients and are correlated with poor overall survival outcomes. However, we also found that the results of the 95% confidence intervals for hazard ratio (HR) of hsa-miR-2681-3p and hsa-miR-6807-3p were inconsistent, the differences may be due to the expression levels of these two miRNAs were zero in some patients, resulting in uneven distribution in the division of high and low expression levels by median. A study found that hsa-miR-379-3p levels in gastric cancer tissue samples were down-regulated, which is consistent with our results [14]. Hsa-miR-6807-3p has been identified to promote glioma tumorigenesis by regulating downstream DACH1 and it can also promote the development of lung cancer through the miR-6807-3p/DKK1 axis [15]. In addition, hsa-miR-6499 has been found to be a potential candidates for counteracting age-related macular degeneration and neurodegenerative diseases [16]. These results further confirmed the reliability of the miRNA prognostic model we established.

After the target genes predicted by online databases are analyzed by GO, we found the results are mainly related to the function of the nervous system, this may be due to the existence of the stomach-brain axis, food intake will activate specific areas of the brain such as thalamus and amygdala. Also, psychological factors and cognitive processes play a role in gastrointestinal disorders [17]. KEGG pathway analysis revealed ultimate target genes were concentrated in TGF-beta pathway and FoxO pathway. The former has tumor suppressor function on early cancer cells, however, activation of it in advanced cancer can promote tumorigenesis [18], it mainly plays various roles through TGF-β/SMAD4 signaling. FoxO is also involved in the regulation and regulation of various biological activities such as development, cell signal transduction and tumorigenesis, and play an important role. It is mainly regulated by the phosphorylation of PI3K/AKT and PKA pathways, is also subjected by ubiquitination, acetylation, and methylation. Therefore, these target genes can also provide new directions for future tumor treatment, especially the five genes *DLC1*, *LRFN5*, *NOVA1*, *POU3F2* and *PRICKLE2* which were positively related to the overall survival time.

The main purpose of our research is to explore new miRNA biomarkers to predict the prognosis of STAD patients. The novelty of this study is that after we obtained the miRNA markers, we also obtained predicted miRNA target genes from online database and verified them by survival analysis and conducted enrichment analysis. These target genes may also be potential biomarkers in treatment of STAD. However, our research still has some limitations, we have not verified our results through clinical samples or cell experiments, and further experiments are still needed to study and verify our results, to improve the reliability of our results. In addition, with the advancement of interaction prediction research in various fields of computational biology [19], the interactions between genetic markers and nc RNAs have also gradually attracted the attention of scientists, such as miRNA-lncRNA interaction prediction [20, 21] and the interactions between circular RNAs and genes [22] and so on. Therefore, we also consider exploring the role of miRNA and nc RNA in STAD and seeking more experimental evidence in the future.

## Conclusions

In this study, we used bioinformatics methods to screen new prognostic miRNA markers from TCGA and established a prognostic model of STAD, so as to provide a basis for the diagnosis, prognosis, and treatment of STAD in the future.

## Methods

This study aimed to identify reliable miRNA prognostic models for STAD. The methods adopted in this paper include obtainment of differential expression genes, construction of prognostic model and survival analysis, miRNA targets prediction, validation, enrichment analysis and the construction of protein–protein interaction (PPI) network.

### Data acquisition

The RNA and the isoform miRNA sequencing files of STAD and corresponding clinical information files were acquired from TCGA. Genome annotation file was downloaded from GENCode [23] to identify mRNA sequences within RNA-Seq data. Mature miRNA sequences were annotated by the R package miRBaseVersions.db [24]. Finally, we obtained mRNA sequences from 405 samples (32 normal and 373 tumor), mature miRNA sequences from 489 samples (45 normal and 444 tumor). The clinical information we extracted included age, gender, survival status, survival time, tumor stage, neoplasm histologic grade and TNM classification was shown in Table 2 except survival status and survival time.

**Table 2:** Clinical information characteristics of STAD patients from TCGA

Variables	Alive	Dead	Number of samples
Age			
> 67/ ≤ 67	131/165	46/27	177/192
Gender			
Male/female	176/120	56/17	232/137
Grade			
G1/G2/G3	5/95/196	1/28/44	6/123/240
Stage			
I/II/III/IV	38/109/129/20	9/9/36/19	47/118/165/39
T			
T1/T2/T3/T4	16/62/134/84	1/13/39/20	17/75/173/104
N			
N0/N1/N2/N3	102/80/60/54	15/17/17/24	117/97/77/78
M			
M0/M1	282/14	61/12	343/26

### Obtainment of differential expression genes

After finishing the organization of original mRNA and mature miRNA sequence expression files, the edger package [25] was used to filter the differential expression mRNA and miRNA. The filter criteria were set as FDR < 0.05 and log_2_(Fold Change) ≥ 1 or ≤ -1.

### Prognostic model and survival analysis

The miRNA expression matrix and clinical data were divided into one training set and one test set by the caret package [26] of R-language and the split ratio of training set and testing set was 7:3 according to the previous research [27, 28]. A univariate COX regression was carried out for the training set using the miRNA expression and the clinical information including age, gender, stage, neoplasm histologic grade, T, N and M classification, and p value < 0.01 was as the filter criterion. Multivariate COX analysis was then built to estimate the risk of five candidate variables that have been screened out. According to the results, the risk score of all patients can be calculated by the predict function in R. Then patients were separated into two groups in accordance with the median of their risk scores. ROC curves were used to determine the accuracy of prognostic model. Unpaired t test was used to compare the expression levels and tumor stages of the miRNAs in the two groups (high-risk and low-risk) of training set and testing set, grouping criteria for high and low risk were based on the median of miRNA expression levels and tumor stages. Kaplan–Meier analyses were used to estimate the overall survival of the four candidate miRNA and the log rank tests were used to evaluate the significance of the difference in survival, the four candidate miRNAs were divided into high and low expression groups according to their median expression levels.

### MiRNA target genes analysis

MiRNA target genes were predicted by The Encyclopedia of RNA Interactomes (ENCORI) [29], miRDB [30] and TargetScan [31] database. The intersection of the target genes predicted by the above three databases were taken as potential miRNA target genes. Common genes in both potential miRNA target genes and differential expression mRNAs were selected as the ultimate miRNA target genes. The connections between miRNAs and the ultimate target genes were visualized by Cytoscape software. GO and KEGG pathway enrichment on the ultimate miRNA target genes package were achieved by the clusterProfiler [32] package. Using the STRING database [33], a PPI network was constructed.

## Supplementary Information

**Additional file 1.** **Table S1.** The differently expressed mRNAs of STAD in TCGA. **Table S2.** The differently expressed miRNAs of STAD in TCGA. **Table S3.** The results of univariate COX regression. **Table S4.** Online databases predicted 50 target genes of these four miRNAs. **Table S5.** The results of GO analysis and KEGG analysis of ultimate target genes

## Data Availability

The datasets used and analyzed during the current study are available in the Cancer Genome Atlas (TCGA, https://portal.gdc.cancer.gov/) database. The dataset supporting the conclusions of this article is included within the article and its additional file. R code and R data files for analysis are also available if required.

## Abbreviations

STAD: Stomach adenocarcinoma
non-coding RNA: Nc RNA
TCGA: The cancer genome Atlas
ROC: Receiver operating characteristic
AUC: Area Under Curve
ENCORI: Encyclopedia of RNA Interactomes
GO: Gene Ontology
KEGG: Kyoto Encyclopedia of Genes and Genomes
BP: Biological Process
CC: Cellular Component
MF: Molecular Function
HR: Hazard ratio
PPI: Protein-protein interaction
